# Authors’ Reply: Which Surgical Operations Should be Performed in District Hospitals in East, Central and Southern Africa? Results of a Survey of Regional Clinicians

**DOI:** 10.1007/s00268-020-05895-3

**Published:** 2020-12-06

**Authors:** Zineb Bentounsi, Chris Lavy, Chiara Pittalis, Morgane Clarke, Jean Rizk, Grace Le Drury, Ruairi Brugha, Eric Borgstein, Jakub Gajewski

**Affiliations:** 1 Nuffield Department of Orthopaedics, Rheumatology and Musculoskeletal Sciences, Oxford, UK; 2 Royal College of Surgeons in Ireland, Dublin, Ireland; 3 Department of Surgery, Queen Elizabeth Central Hospital, Blantyre, Malawi

Dear Editor,

We thank Priyanch Nathani et al. for the evidence from India that endorses the importance of providing essential surgery at district hospitals (DHs), following our article [1]. We welcome the analysis linking our survey results with population-based needs for essential surgical operations from India and agree with the need for more evidence-based strategies to help DHs to meet the surgical needs of populations. Our study was the first empirical attempt to define an essential district-level surgical package for East, Central and Southern Africa, which could be replicated in India and other low- and middle-income countries (LMICs).

As highlighted by Nathani et al., more evidence is needed on the reasons behind the low level of positive agreement expressed by clinicians on delivery of a subset of certain operations at DHs, in particular ‘high demand’ procedures. Collaborative work to define the essential surgical package at district level in LMICs should focus on deepening our understanding on how to reach agreement on what package of surgeries is essential in what context. A critical factor in interpreting the Group 2 procedures, where surgeons’ levels of positive agreement ranged from 31 to 79%, is that it includes what are often emergency life-saving major interventions—hysterectomy, splenectomy and bowel resection—where timely (life-saving) referral of patients to a specialist centre is simply not feasible.

This leads to a second critical and sensitive issue: identifying who will perform these operations in DHs, in view of the inability of many countries to place and retain specialist surgeons and anaesthetists in district hospitals. Lessons learned from interventions such as COST-Africa [2] and SURG-Africa [3] have demonstrated how to increase access to safe and effective essential surgery at district level. The tested model uses specialist surgeons and anaesthetists to train, supervise and mentor surgical teams, including non-physician clinicians (NPCs) and general medical officers, to undertake many of these procedures. Task sharing major surgery to DH-based NPCs may raise concerns, which is why we need to further build the evidence base, by demonstrating the safety and effectiveness of these models [4].

Finally, it is essential to prioritise DHs in national funding allocations to different healthcare facilities. We join Nathani et al. in calling international funders who invest in strengthening health systems to prioritise investment in DHs. A way forward to crystallise political will on improving surgical care delivery in DHs is to ensure that empirically demonstrated feasible, safe and effective models of district surgical care are included in National Surgical Obstetrics and Anaesthesia Plan. The surgical needs of rural and district populations must be prioritised if global disease burden goals are to be achieved.
